# Tuberculosis diagnosis cascade in Blantyre, Malawi: a prospective cohort study

**DOI:** 10.1186/s12879-021-05860-y

**Published:** 2021-02-15

**Authors:** Helena R. A. Feasey, Elizabeth L. Corbett, Marriott Nliwasa, Luke Mair, Titus H. Divala, Wala Kamchedzera, Mc Ewen Khundi, Helen E. D. Burchett, Emily L. Webb, Hendramoorthy Maheswaran, S. Bertel Squire, Peter MacPherson

**Affiliations:** 1 Malawi-Liverpool-Wellcome Trust Clinical Research Programme, Blantyre, Malawi; 2 London School of Hygiene & Tropical Medicine, Keppel Street, Bloomsbury, London, UK; 3 Helse Nord Tuberculosis Initiative, College of Medicine, University of Malawi, Blantyre, Malawi; 4 Liverpool School of Tropical Medicine, Liverpool, UK; 5 University of Liverpool, Liverpool, UK

## Abstract

**Background:**

Tuberculosis (TB) control relies on early diagnosis and treatment. International guidelines recommend systematic TB screening at health facilities, but implementation is challenging. We investigated completion of recommended TB screening steps in Blantyre, Malawi.

**Methods:**

A prospective cohort recruited adult outpatients attending Bangwe primary clinic. Entry interviews were linked to exit interviews. The proportion of participants progressing through each step of the diagnostic pathway were estimated. Factors associated with request for sputum were investigated using multivariable logistic regression.

**Results:**

Of 5442 clinic attendances 2397 (44%) had exit interviews. In clinically indicated participants (*n* = 445) 256 (57.5%) were asked about cough, 36 (8.1%) were asked for sputum, 21 (4.7%) gave sputum and 1 (0.2%) received same-day results. Significant associations with request for sputum were: any TB symptom (aOR:3.20, 95%CI:2.02–5.06), increasing age (aOR:1.02, 95%CI:1.01–1.04 per year) and for HIV-negative participants only, a history of previous TB (aOR:3.37, 95%CI:1.45–7.81). Numbers requiring sputum tests (26/day) outnumbered diagnostic capacity (8–12/day).

**Conclusions:**

Patients were lost at every stage of the TB care cascade, with same day sputum submission following all steps of the diagnosis cascade achieved in only 4.7% if clinically indicated. Infection control strategies should be implemented, with reporting on early steps of the TB care cascade formalised. High-throughput screening interventions, such as digital CXR, that can achieve same-day TB diagnosis are urgently needed to meet WHO End TB goals.

**Supplementary Information:**

The online version contains supplementary material available at 10.1186/s12879-021-05860-y.

## Background

Tuberculosis (TB) is the leading infectious cause of death worldwide and an estimated 10 million people developed TB disease in 2019 [1, 2]. TB control relies on early diagnosis and treatment, as reflected in the World Health Organization (WHO) End TB 2025 target of ≥90% of people who develop TB being notified and treated [3]. To achieve this the WHO recommends systematic TB screening for priority risk groups in order to reduce poor disease outcomes and TB transmission [4]. These recommendations are reflected in many National TB Programme (NTP) guidelines [5–8].

The TB care cascade model assesses patient progression and retention through sequential stages of care required to achieve a successful treatment outcome [9], in order to quantify gaps in care delivery and adherence to guidelines. Care cascades have been extensively used to evaluate HIV care delivery [10], but have only recently been applied by TB programmes [9] to expand analysis beyond standardised treatment outcome reporting [11] and ad hoc diagnostic pathway analysis [12].

In Subbaraman et al’s generic model for a care cascade for active TB the first gap is identified as not accessing a TB diagnostic test [9]. This first gap is repeatedly the largest in many settings [13, 14], in keeping with the numerous issues relating to sputum-based tests [15].

Recent studies have emphasised variability in TB diagnosis cascades in high burden countries. In India, only 12–17% of standardised patients were correctly asked to test for TB [16], whereas in Nairobi, Kenya [17] this was 50% and a systematic review found a range for all patients from 4% in Thailand to 84% in South Africa [18]. However, most high-burden TB countries, do not routinely collect data to estimate adherence to systematic TB screening guidelines in health facilities.

WHO recommends people living with HIV are systematically screened for TB each time they visit a health facility and that in high-burden TB settings systematic screening for TB in other selected high risk groups may also be appropriate [4]. These risk groups include older people and those previously treated for TB. However, an estimated 29% of new TB cases are still not identified or officially notified, partly due to failure to diagnose active TB in people accessing healthcare [1, 19]. Examining TB test access for these risk groups and subsequent steps in the TB diagnosis cascade will be critical for efficient TB programme design.

The aims of this study were to: construct a TB diagnosis care cascade; describe the proportion of “clinically-indicated” patients (defined by the Malawi National guidelines [5]) who progressed through each step of the diagnosis cascade in a primary care clinic; and investigate factors associated with being offered a TB test.

## Methods

### Study design

A prospective cohort of adults aged 18 years and older was recruited from May to September 2018. The study formed part of the pilot phase of a randomised trial at Bangwe health clinic in Blantyre, Malawi [20].

### Study site and population

Patients self-presenting to free-of-charge acute-care services in Bangwe Health Centre – a government primary care clinic – were recruited prospectively. There are no physicians at the clinic; care is provided by nurses and clinical officers, who conduct consultations, including TB symptom screening, with the patients. There is a GeneXpert machine for TB sputum diagnosis and TB treatment is available on site. TB prevalence in Blantyre was 1% in 2013 [21] and 113 new registrations for TB treatment were recorded at Bangwe health centre during 2018 (unpublished data).

Malawi National TB Programme guidelines state that all adults with HIV presenting to healthcare facilities with any TB symptom (any of cough, night sweats, fever or weight loss) should receive a sputum test for TB [5]. For HIV-negative adults sputum tests are recommended for all those with weight loss or other TB symptoms of two weeks or more. For the purposes of this study ‘clinically indicated to submit sputum’ was defined as adults with HIV with any TB symptom and HIV-negative adults with weight loss or a chronic cough (two weeks or more), since duration data was not collected for night sweats or fever. A sensitivity analysis was conducted with an alternative definition including any symptom of any duration for people without HIV infection.

### Data collection

Research assistants stationed at the registration desk in the acute-care clinic asked all patients for verbal consent to participate. A fingerprint scan with demographic details was recorded electronically at entry interview. Additional research assistants positioned by the two clinic exits asked all adults leaving the clinic to participate in exit interviews. Participants provided written or witnessed fingerprint (if illiterate) consent for exit interviews.

Entry and exit interviews were linked through digital fingerprint bio-identification. Entry interviews recorded age, sex and WHO recommended TB symptom screening [4, 22]. Exit interviews asked about care received at the clinic and included self-reported HIV status and previous TB diagnosis; whether a health worker had enquired about cough; if they had been asked to submit sputum; if they submitted sputum; and if sputum results had been received. For simplicity, the exit interview enquiry about symptom screening referred only to cough, as this is the most commonly recognised TB symptom in Malawi [23]. Questionnaires were kept brief to minimise inconvenience and maximise the completeness of capture (see Suppl Table 2 for full questionnaires).

### Statistical methods

Summary statistics compared characteristics (collected at clinic entry) of participants who had exit interviews with those who had not (***χ***^**2**^ and Kruskal-Wallis tests). Participant characteristics were also compared by HIV status (HIV-positive, HIV-negative, status unknown/never tested). “Chronic cough” was defined as cough ≥2 weeks. “Any TB symptom” included any reported cough, fever, weight loss or night sweats [24].

Diagnosis care cascades were constructed based on all participants, and separately for clinically-indicated groups: HIV-negative participants with weight loss or chronic cough and people living with HIV (PLHIV) with any TB symptom. Generic care cascade Step 2 ‘Accessed TB tests’ [9] was expanded to explore symptom enquiry (cough); request to submit sputum; and sputum submission.

Univariable and multivariable logistic regression were used to investigate associations of clinical and demographic characteristics with request for sputum submission. Separate models were fit for ‘any TB symptom’ and specific individual TB symptoms. Sex, age and symptom variables included in the Malawi Tuberculosis Guideline [5] were included a priori in the multivariable models.

Those who reported a cough and being on TB treatment or isoniazid preventive therapy (IPT) at clinic entry were removed from the cascade and multivariable analysis.

### Ethical considerations

Approval was received from the research ethics committees of the College of Medicine, Malawi and Liverpool School of Tropical Medicine. All participants provided written informed consent (or witnessed, thumb-print consent if illiterate).

### Data and reproducibility

Data and code to reproduce this analysis is available from *https://github.com/petermacp/tbcascade**.*

## Results

### Clinic attendee characteristics

**Fig. 1:**
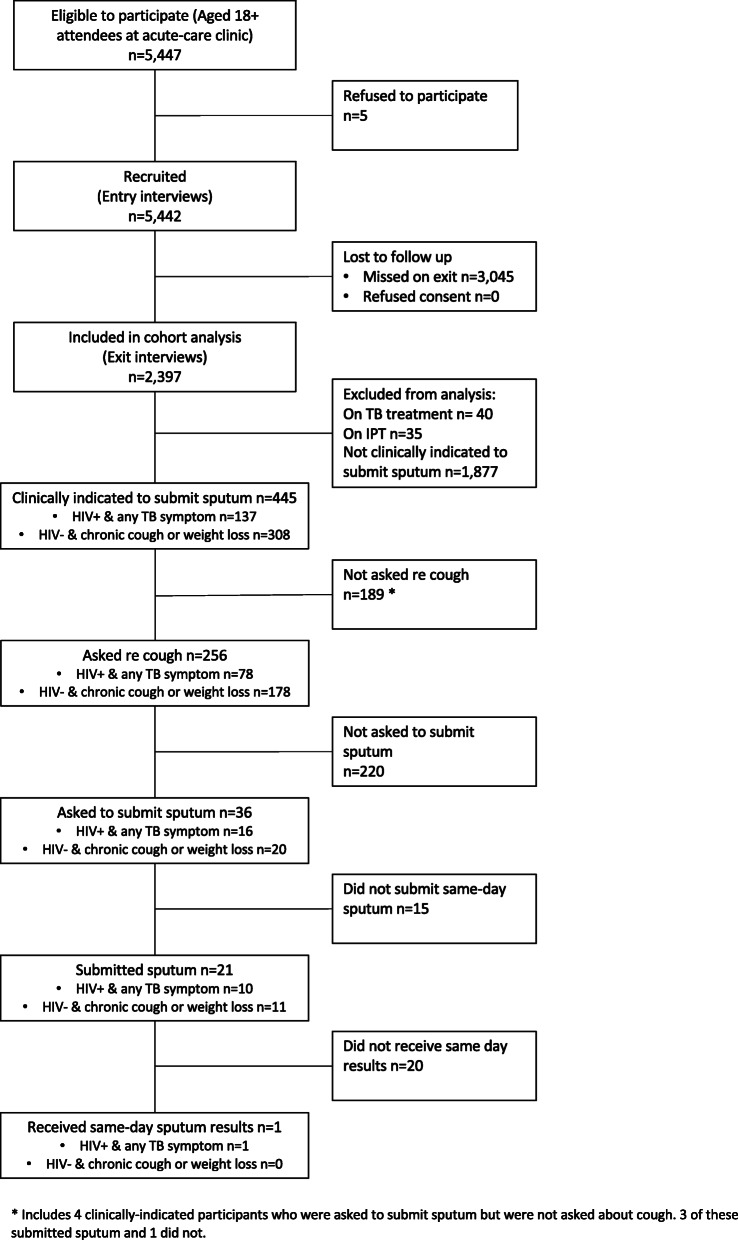
Consort diagram of cohort participants and progress through steps of the TB diagnosis cascade

Of 5442 clinic attendances 2397 (44%) had matched exit interviews, mainly reflecting limited study capacity to interview everyone leaving the clinic (Fig. 1). Five individuals declined to participate in entry interviews and were not included in the study. None refused to participate in exit interviews.

Participants with matched exit interviews had similar characteristics to those with just an entry interview, with some differences: men were more likely to complete an exit interview (37.5% vs 34.2%, *p* = 0.012) as were those with any TB symptom (57.2% vs 54.4%, *p* = 0.044). This was consistent for cough, fever and night sweats. Those completing exit interviews were older than those who did not (median age 28 vs 27 years) (Suppl Table 1).

**Table 1:** Baseline characteristics of exit interview participants by HIV status

	HIV+ (***N*** = 292)	HIV- (***N*** = 1809)	Don’t know/never tested (***N*** = 296)	***P*** value
Sex (Female)	213 (72.9%)	1134 (62.7%)	150 (50.7%)	< 0.001
Age Median (Range)	36 (18–70)	27 (18–87)	27 (18–89)	< 0.001
Age 18–29	89 (30.5%)	1031 (57.0%)	159 (53.7%)	< 0.001
30–39	98 (33.6%)	391 (21.6%)	46 (15.5%)	
40–49	68 (23.3%)	190 (10.5%)	25 (8.4%)	
50–59	22 (7.5%)	95 (5.3%)	27 (9.1%)	
60–89	15 (5.1%)	102 (5.6%)	39 (13.2%)	
Cough	121 (41.4%)	618 (34.2%)	110 (37.2%)	0.044
Cough days (if cough) Median (Range)	7 (1–3650)	4 (1–2190)	4 (2–1095)	0.001
Chronic cough^¶^	44 (15.1%)	149 (8.2%)	28 (9.5%)	0.001
Weight loss	65 (22.3%)	223 (12.3%)	28 (9.5%)	< 0.001
Fever	92 (31.5%)	550 (30.4%)	92 (31.1%)	0.915
Night sweats	56 (19.2%)	342 (18.9%)	63 (21.3%)	0.629
Any symptoms^†^	181 (62.0%)	1007 (55.7%)	182 (61.5%)	0.035
Previous TB	66 (22.6%)	66 (3.6%)	9 (3.0%)	< 0.001
On TB treatment*	18 (14.9%)	20 (3.2%)	2 (1.8%)	< 0.001
TB treatment last 6 months*	2 (1.7%)	7 (1.1%)	0 (0.0%)	0.446
On IPT*	26 (21.5%)	7 (1.1%)	2 (1.8%)	< 0.001
ART	276 (94.5%)	0 (0%)	0 (0%)	< 0.001
Self-reported general health				
Very Good	5 (1.7%)	58 (3.2%)	13 (4.4%)	0.062
Good	159 (54.5%)	1091 (60.3%)	159 (53.7%)	
Fair	122 (41.8%)	622 (34.4%)	119 (40.2%)	
Poor/Very poor	6 (2.1%)	38 (2.1%)	5 (1.7%)	

† Any TB symptom: cough, or weight loss, or fever, or weight loss

¶ Cough of 14 days or longer

* Only recorded if patient had cough

### Exit interviewee characteristics

Of the 2397 with matched exit interviews 900 (37.5%) were male. Median age was 28 years (range 18–89). A total of 849 (35.4%) had a cough, with 221 (9.2%) having chronic cough, and 1370 (57.2%) having any TB symptom. Previous TB treatment was reported by 141 (5.9%). Among HIV positive participants (292, 12.2%) almost all were taking antiretroviral therapy (ART) (276, 94.5%). Of those completing exit interviews 1485 (62.0%) self-reported good health.

HIV positive participants were more likely than HIV-negative or status-unknown participants to be female (72.9% vs 62.7 and 50.7%, *p* < 0.001) and older (median age 36 years vs 27 years and 27.5 years for HIV-positive, HIV-negative and HIV-unknown respectively, p < 0.001) (Table 1). PLHIV were also more likely to be taking TB treatment (14.9% vs 3.2 and 1.8%), on IPT (21.5% vs 1.1 and 1.8%) and to report previous TB (22.6% vs 3.6 and 3.0% for HIV-positive, HIV-negative and HIV-unknown respectively) (all p < 0.001). A higher proportion of PLHIV had chronic cough (15.1%) compared to HIV-negative (8.2%) or unknown-status participants (9.5%, *p* = 0.001).

75 participants who reported being on TB treatment (40 people) or isoniazid preventive therapy (IPT) (35 people) were not included in the cascade or multivariable analysis.

### TB diagnosis cascades

Of all 2322 exit interview participants analysed 1322 (56.9%) were asked by health workers about cough, 118 (5.1%) were asked to submit sputum, 46 (2.0%) gave same-day sputum and 3 (0.1%) received same-day results.

445 participants were clinically-indicated to submit sputum (HIV-negative participants with weight loss or chronic cough, and PLHIV with any TB symptom). 256 (57.5%) of these reported having been directly asked about coughing, with 36 of those (36/445, 8.1% of total) asked to submit a sputum sample; 21/445 (4.7%) provided same-day sputum and 1/445 (0.2%) received same-day sputum results (Fig. 1).

**Fig. 2:**
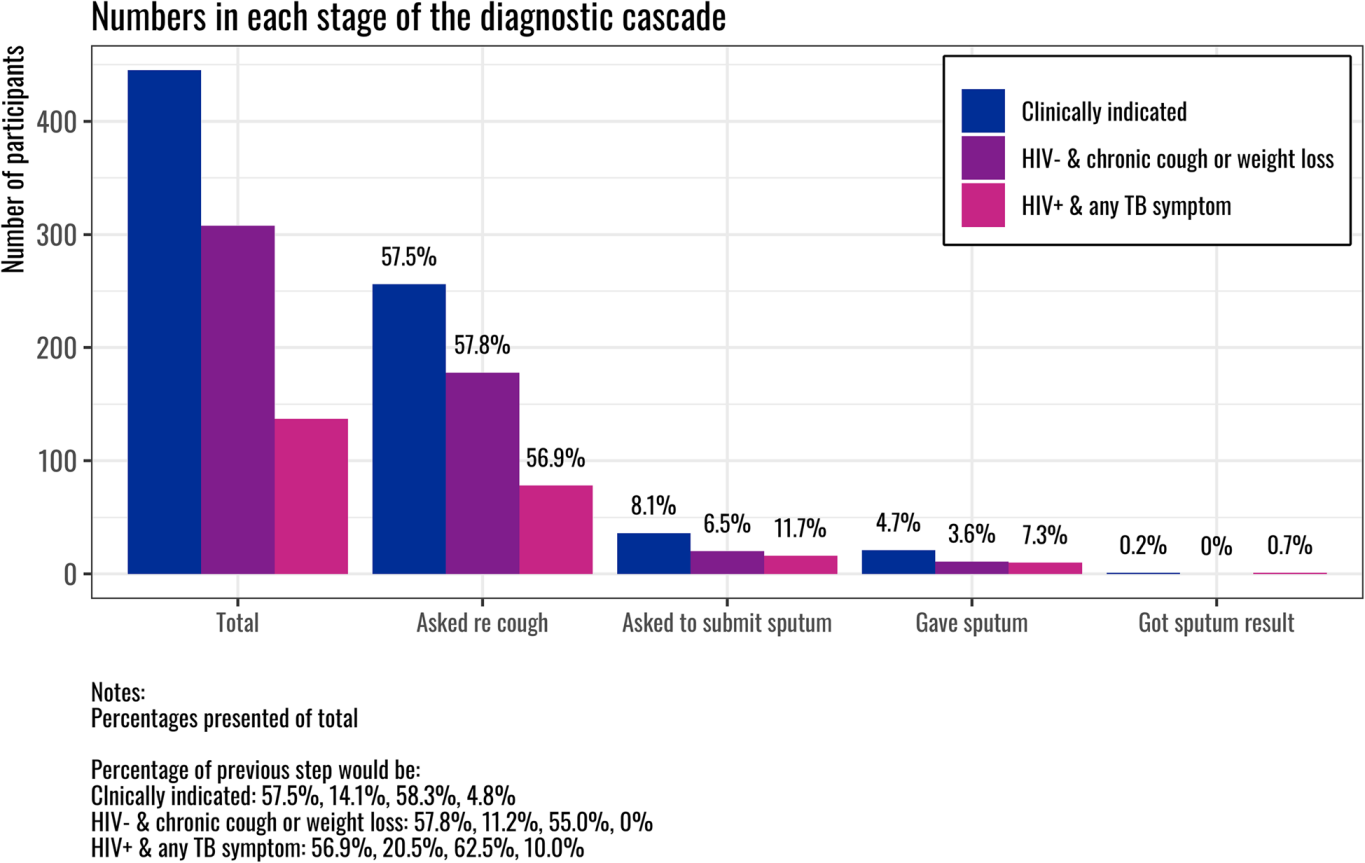
Diagnosis care cascades for groups clinically indicated for TB sputum tests

Diagnosis care cascades were constructed separately for each clinically-indicated group: HIV-negative participants with weight loss or chronic cough and PLHIV with any TB symptom (Fig. 2). In the 308 HIV-negative participants with chronic cough, 57.8% were asked about cough, 6.5% were also asked for sputum, 3.6% gave sputum and none received same-day results. Among the 137 PLHIV with any TB symptom 56.9% were asked about cough, 11.7% were also asked for sputum, 7.3% gave sputum and 0.7% received same-day results. Overall sputum submission for TB testing was achieved in 5.4% (24/445) of clinically-indicated participants with 4.7% (21/445) successfully progressing through all steps of the diagnosis cascade to this point (four clinically indicated participants were requested to give sputum but had not been asked about cough).

Of the 118 requested to submit sputum 78 (66.1%) were not classed as clinically indicated. Of these 10/78 (12.8%) had unknown HIV status and TB symptoms, 27/78 (34.6%) were HIV-negative with no chronic cough or weight loss but had night sweats or fever of unknown duration and 17/78 (21.8%) were HIV-negative with cough of < 2 weeks as their only TB symptom. 24/78 (30.8%) participants (of whom 2 were HIV-positive) reported being asked to submit sputum but did not report any TB symptoms.

Using a more sensitive definition for clinically indicated participants (HIV-positive with any TB symptom and HIV-negative with chronic cough or weight loss, night sweats or fever of any duration) reduced the proportion of clinically indicated participants asked to submit sputum to 5.7% (8.1% in original definition) and those who submitted sputum to 2.4% (4.7% originally).

For all clinically-indicated groups, the biggest gap in the diagnosis cascade was between symptom enquiry and requesting sputum with 49.4% (220 of 445 who were clinically indicated for sputum testing) lost at this stage compared to 42.5% (189/445) not asked about cough and 3.4% (15/445) not giving sputum despite health worker request. For HIV-negative participants with weight loss or chronic cough, clinicians requested sputum for 11.2% (20/178) of those they had asked about cough and in PLHIV with any TB symptom this was 20.5% (16/78).

### Factors associated with being asked to submit sputum

On univariable analysis for all participants (Table 2), factors significantly associated with being asked to submit sputum included: older age (OR: 1.02, 95%CI: 1.01–1.03 per year increase in age), previous TB treatment (OR: 2.13, 95%CI: 1.08–4.20); being HIV-positive (OR: 1.69, 95%CI: 1.02–2.80); and presence of any TB symptoms (cough< 2 weeks OR: 2.48 (95%CI: 1.70–3.61), chronic cough OR: 3.32 (95%CI:2.07–5.33), weight loss OR: 2.52 (95% CI: 1.63–3.89), fever OR 2.10 (95% CI: 1.44–3.06) and night sweats OR 1.86 (95%CI: 1.23–2.80)).

**Table 2:** Univariable and multivariable associations with being asked to submit sputum: all participants. *n* = 2322

Variable	Unadjusted OR		Adjusted OR Any TB symptom		Adjusted OR Individual symptoms	
	OR (95% CI)	P value	aOR (95% CI)	P value	aOR (95% CI)	P value
Sex	1.02 (0.69–1.49)	0.936	1.01 (0.68–1.49)	0.975	1.08 (0.73–1.62)	0.695
Age	1.02 (1.01–1.03)	< 0.001	1.02 (1.01–1.04)	< 0.001	1.02 (1.01–1.03)	0.002
Previous TB	2.13 (1.08–4.20)	0.026	1.64 (0.79–3.37)	0.183	1.59 (0.75–3.37)	0.224
HIV + *	1.69 (1.02–2.80)	0.040	1.42 (0.84–2.42)	0.191	1.45 (0.85–2.49)	0.174
Any TB symptom†	3.27 (2.07–5.18)	< 0.001	3.20 (2.02–5.06)	< 0.001	–	–
Cough < 2 weeks	2.48 (1.70–3.61)	< 0.001	–	–	3.43 (2.23–5.28)	< 0.001
Chronic cough	3.32 (2.07–5.33)	< 0.001	–	–	3.71 (2.10–6.56)	< 0.001
Weight loss	2.52 (1.63–3.89)	< 0.001	–	–	1.54 (0.96–2.47)	0.076
Fever	2.10 (1.44–3.06)	< 0.001	–	–	1.43 (0.94–2.18)	0.096
Night sweats	1.86 (1.23–2.80)	0.003	–	–	1.05 (0.66–1.68)	0.827

* Reference group: HIV-negative. Status unknown not presented

† Any TB symptom: cough, or weight loss, or fever, or weight loss

¶ Cough of 14 days or longer

On multivariable analysis increasing age (adjusted OR: 1.02, 95%CI: 1.01–1.04 per year) and any TB symptom (adjusted OR: 3.20, 95%CI: 2.02–5.06) remained significantly associated with being asked to submit sputum for all participants. In the individual symptoms multivariable model presence of cough (both under and over 2 weeks duration) was the only symptom still significantly associated with request for sputum (cough< 2 weeks adjusted OR 3.43, 95%CI: 2.23–5.28, chronic (≥2 weeks) cough adjusted OR: 3.71, 95%CI: 2.10–6.56) (Table 2).

On stratification by HIV status all these factors remained significantly associated for HIV-negative participants, but only the presence of any TB symptom (OR: 8.24, 95%CI: 1.08–37.68, adjusted OR: 8.18, 95%CI: 1.85–36.21) and chronic cough (OR: 10.84, 95%CI: 3.66–32.09, adjusted OR: 13.06, 95%CI: 3.69–46.28) were significantly associated with request for sputum amongst PLHIV (Table 3).

**Table 3:** Univariable and multivariable associations with being asked to submit sputum by HIV status

	HIV-positive ***n*** = 248				HIV-negative ***n*** = 1782			
	Univariable		Multivariable		Univariable		Multivariable	
	OR (95% CI)	P value	aOR (95% CI)	P value	OR (95% CI)	P value	aOR (95% CI)	P value
Sex	1.15 (0.40–3.29)	0.801	1.08 (0.35–3.32)	0.891	1.04 (0.67–1.62)	0.867	1.18 (0.74–1.87)	0.485
Age	1.02 (0.98–1.06)	0.394	1.02 (0.98–1.06)	0.383	1.02 (1.01–1.04)	0.001	1.02 (1.01–1.04)	0.008
Previous TB	0.51 (0.11–2.28)	0.367	0.52 (0.11–2.48)	0.413	3.66 (1.67–8.06)	0.001	3.37 (1.45–7.81)	0.005
Any TB symptom†	8.24 (1.80–37.68)	0.001	8.18 (1.85–36.21)	0.006	2.56 (1.56–4.20)	< 0.001	-*	-*
Cough < 2 weeks	1.28 (0.44–3.72)	0.645	-*	-*	2.59 (1.68–4.01)	< 0.001	3.16 (1.94–5.13)	< 0.001
Chronic cough¶	10.84 (3.66–32.09)	< 0.001	-*	-*	2.37 (1.30–4.33)	0.004	2.56 (1.25–5.25)	0.010

† Any TB symptom: cough, or weight loss, or fever, or weight loss

¶ Cough of 14 days or longer

* Multivariable analysis for HIV+ presented for Any TB symptom model, for HIV- presented model includes individual symptoms. Other symptoms (weight loss, fever and night sweats included in model but not presented: no significant relationship on multivariate analysis)

### Sputum test throughput and capacity

If all patients clinically indicated for a TB test did submit sputum (445/44% [percentage completing exit interview] = 1011 over the 78 working days of the study) that would result in ~ 26 sputum samples on each working day (13 patients a day, each with two samples). The clinic laboratory has one GeneXpert machine to process TB samples, with a maximum throughput of 8–12 samples a day (4 samples per cartridge with 2 h run time plus preparation).

## Discussion

This study found that same day sputum submission for TB testing following all steps of the diagnosis cascade was achieved for only 4.7% of participants among whom sputum testing was indicated according to Malawi national guidelines, with patients lost at every stage of the TB diagnosis care cascade. Failure to request sputum by clinicians despite elicited symptoms led to the biggest single gap in the diagnosis care cascade, followed by not asking about symptoms. This suggests that: interventions focusing on health worker behaviour may have the greatest potential for retaining presumptive TB patients within the diagnosis cascade; there appears to be inconsistent application of guidelines and infection control practices; and that we must formalise and strengthen reporting on the early steps in the TB care cascade. Additional important epidemiological groups such as men [1] should be given equal priority to PLHIV within national TB guidelines. However, if guideline adherence is improved, novel high-throughput triage testing approaches will also be needed to reach the required capacity.

Adherence to sputum-request guidelines in 4.7% (21/445) of patients sits at the bottom of the range (4–84%) identified in a recent systematic review [18]. When taken together with a TB treatment initiation rate of 85–94% [25] and TB treatment success rate of 82% in Malawi [19], our data suggests that the overall TB cascade in Malawi is more similar to that for India than that for South Africa. In India gap 1 (did not access a TB diagnostic test) accounted for 50% of all patient losses, whereas in South Africa, low treatment success led to the largest gap in the cascade [9, 13, 16].

To reduce these substantial gaps in accessing TB tests a multi-faceted approach is required to identify logistical barriers and change health worker behaviours. Facility-based screening relies on health worker behaviour (asking about symptoms and requesting sputum) which leads to the biggest gaps and therefore offers the greatest potential for improvement. Suspicion of malaria or bacterial investigations may contribute to not requesting sputum [26] but further investigation is needed to confirm what structural factors drive health worker behaviour.

This study demonstrates a low level of adherence to National TB Programme guidelines. This is the case even with groups identified as high risk within both the Malawi and WHO guidelines, such as those who have previously had TB and PLHIV. Health workers operate in challenging conditions with average patient consultation times < 3 min [27], a high turnover of staff and regular supply stock outs [28]. As such, measures undertaken to improve adherence to guidelines and increase the proportion of clinically-indicated patients who access TB tests need to be pragmatic. Strategies such as FAST - Finding TB cases Actively, Separating safely and Treating effectively – [29] are effective in increasing testing and infection control not only for TB but also other respiratory infections. In Malawi, some elements of FAST, such as cough monitors, have been inconsistently implemented, due to limited availability of resources. However, our analysis shows the large gap in cough and symptom enquiry that could be met by universal cough monitors. Implementing strategies such as FAST consistently is critical for all low and middle income countries (LMICs), especially in the midst of the COVID-19 pandemic.

In addition, enhanced monitoring and central collation of data are essential to tracking individual clinic performance. Malawi, as is typical for LMICs, collects and reports comprehensive data on TB case notification and treatment success at clinic level, but only reports the number of TB tests per facility per quarter, without further diagnostic steps. A WHO recommendation to report numbers of screened presumptive TB cases, disaggregated by age, gender and HIV-status globally, would allow greater focus on the earlier steps of the TB care cascade.

Despite TB prevalence in men being over twice as high as among women in LMICs [30] and in Malawi a ratio of male to female cases of 1.5 [1, 19], in our study sex was not associated with being requested to submit sputum. In Malawi, the ratio of prevalent-to-notified cases of TB – an indication of how long patients take to be diagnosed - is 1.5 times higher among men than women [30]. Men should, therefore, be considered as much of a priority group within TB guidelines as PLHIV in countries with a high male-to-female case ratio. Notably, men are less likely than women to seek health care early on in their illness [31], making it critical to manage them efficiently when they do present to a facility.

Finally, if all patients attending the outpatient clinic were screened for TB as per the guidelines, the current Xpert facilities would only be able to process up to a half of the required samples. It is unknown to what extent this lack of diagnostic capacity may influence test decisions among the health workers. If guideline adherence and increased identification of presumptive TB patients is subsequently improved a novel high-throughput approach to triage testing using new diagnostics (e.g. computer aided diagnostics for X-rays) will also be required for LMICs to increase capacity [32, 33].

Study limitations include the single site nature of this study, limiting generalisability, although the study site is typical of urban primary care clinics in Malawi so likely representative of primary care in the areas with highest TB burden in the country [21]. Due to limited research staff capacity we interviewed only 44% of clinic attendees with men and those with TB symptoms more likely to complete an exit interview (Suppl Table 1), potentially resulting in selection bias and overestimation of the proportion who are clinically-indicated. However, this is mitigated by high participation in those approached. Symptoms, HIV status and testing practices were self-reported, potentially resulting in social desirability bias in measurement of these variables, with HIV-positive status under-reported by up to 40% in Malawi [34] and extensive dual HIV and TB stigma [35]. Although healthcare workers would be dependent on the same self-report of TB symptoms to assess eligibility for sputum testing and the proportions progressing through each step of the diagnosis cascade were similar for all HIV status groups. In addition, 69.5% of patients asked to submit sputum were not clinically-indicated to do so as per our definition and 30.8% of those had no reported TB symptoms at all – it is unknown why sputum was requested from these patients.

## Conclusion

Same-day sputum submission for TB testing following all steps of the diagnosis cascade was achieved in only 4.7% of those clinically indicated. Requesting sputum after eliciting symptoms is the key point of the cascade to intervene. Interventions are needed to optimise TB screening guidelines, formalise reporting, increase guideline adherence and improve diagnostic capacity, in order to reduce the most significant gaps early in the TB care cascade and to reach the required testing capacity to meet the WHO End TB goals.

## Supplementary Information

**Additional file 1 Table S1.** Characteristics of adult acute clinic attendances by exit interview participation.

**Additional file 2.**

## Data Availability

The dataset supporting the conclusions of this article is available in the Github repository, *https://github.com/petermacp/tbcascade**.*

## Abbreviations

TB: Tuberculosis
WHO: World Health Organisation
NTP: National TB Programme
PLHIV: People living with HIV
IPT: Isoniazid preventive therapy
CI: Confidence Interval
OR: Odds Ratio
aOR: Adjusted Odds Ratio
FAST: Finding TB cases Actively, Separating safely and Treating effectively
LMICs: Low and middle income countries
