# Supplementary material for: Tuberculosis diagnosis cascade in Blantyre, Malawi: a prospective cohort study
Source: BMC Infect Dis. 2021 Feb 15;21:178. doi: 10.1186/s12879-021-05860-y (PMC7883960; doi:10.1186/s12879-021-05860-y)
Supplement: Supplementary file 1 — Additional file 1 Table S1. Characteristics of adult acute clinic attendances by exit interview participation. [file 12879_2021_5860_MOESM1_ESM.docx]

**Supplemental Table 1:** **Characteristics of adult acute clinic attendances by exit interview participation**

|  | **Exit Interviewed (N=2397)** | **Not exit interviewed (N=3025)** | **Total (N=5422)** | **P value** |
| --- | --- | --- | --- | --- |
| Sex |  |  |  | 0.012 |
| Female | 1497 (62.5%) | 1989 (65.8%) | 3486 (64.3%) |  |
| Male | 900 (37.5%) | 1036 (34.2%) | 1936 (35.7%) |  |
| Age |  |  |  |  |
| Median (Range) | 28 (18, 89) | 27 (18, 89) | 28 (18, 89) | 0.001 |
| Cough |  |  |  |  |
| No | 1548 (64.6%) | 2053 (67.9%) | 3601 (66.4%) | 0.011 |
| Yes | 849 (35.4%) | 972 (32.1%) | 1821 (33.6%) |  |
| Weight loss |  |  |  |  |
| No | 2081 (86.8%) | 2629 (86.9%) | 4710 (86.9%) | 0.920 |
| Yes | 316 (13.2%) | 396 (13.1%) | 712 (13.1%) |  |
| Fever |  |  |  |  |
| No | 1663 (69.4%) | 2178 (72.0%) | 3841 (70.8%) | 0.035 |
| Yes | 734 (30.6%) | 847 (28.0%) | 1581 (29.2%) |  |
| Night sweats |  |  |  |  |
| No | 1936 (80.8%) | 2502 (82.7%) | 4438 (81.9%) | 0.065 |
| Yes | 461 (19.2%) | 523 (17.3%) | 984 (18.1%) |  |
| Any symptoms^†^ |  |  |  |  |
| No | 1027 (42.8%) | 1379 (45.6%) | 2406 (44.4%) | 0.044 |
| Yes | 1370 (57.2%) | 1646 (54.4%) | 3016 (55.6%) |  |
| Chronic cough^¶^ |  |  |  |  |
| No | 2176 (90.8%) | 2724 (90.1%) | 4900 (90.4%) | 0.365 |
| Yes | 221 (9.2%) | 301 (9.9%) | 522 (9.6%) |  |

† Any TB symptom: cough, or weight loss, or fever, or weight loss.
¶ Cough of 14 days or longer
